# Transactions of the Medical Society of the County of Kings

**Published:** 1865-03

**Authors:** 


BUFFALO

### Medical and Surgical Journal.

VOL. IV. MARCH, 1865. No. 8.

ART. I.—*Transactions of the Medical Society of the County of Kings.*

REGULAR MEETING, APRIL, 1863.

*Amputation of the Leg.*

DR. WM. GILFILLAN showed a leg which he had amputated above the knee. The subject was a child, who had been run over that afternoon by a railroad car, producing a *comminuted fracture* of the leg, with great destruction of the soft parts. In this instance there was very little hemorrhage; the *posterior tibial artery* was torn across, but the child did not seem to suffer from the shock. The operation was performed three hours after the accident.

DR. ENOS thought it a very unusual circumstance, that there was no *shock* to the system after such a severe injury.

DR. GILFILLAN thought it might be owing to the sudden and complete destruction of the parts.

*Disarticulation of the Wrist.*

DR. ENOS reported a case of *disarticulation of the hand at the wrist*, from injuries produced by a *hay-cutting machine.* In this case the styloid process of the radius, and not that of the ulna, was removed in order to give the patient the power of *pronation* and *supination.* There was no shock to the system, and but slight evidence of pain. No anæsthetic was used.

*Aneurism of the Aorta.*

DR. SPEIR presented a specimen of *aneurism* at the *arch* of the *aorta.* The subject, a seaman, was admitted into the Brooklyn City Hospital on November 17th, 1862, for aneurism, which was discovered two years previously, while an inmate of that institution. The prominent symptoms were, palpitation, great dyspnœa, vertigo, hoarseness of voice, constant beating pains on the right side of the neck, occasionally shooting down the right arm; the patient lies upon the left side, spits up a thick mucus, mixed with dark, thick blood; the left radial pulse almost imperceptible; the left brachial, subclavian and carotid arteries pulsate less forcibly than natural; increased pulsation and *thrill* in the right subclavian and carotid arteries, with *aortic direct* and *regurgitant murmur.*—The patient died on the 19th of April, 1862.

The post mortem revealed a large amount of serous fluid in the pleural cavity; the heart was double its normal size, and the whole arch of the aorta was very much dilated; the kidneys presented the usual appearance of Bright's Disease, and there was a fatty degeneration of the liver. There were two other aneurismal *tumors* connected with the subclavian and carotid arteries, one and one-half by one inch, which were completely blocked up with fibrin. In one of these tumors the pneumogastric nerve was almost obliterated.

Dr. Bell stated that he had had charge of this patient for two months. He deemed it somewhat remarkable that no absorption of bone had taken place. The disease seemed to have terminated earlier than is usual in similar cases, and he thought it was, probably owing to interference with respiration, the life of the patient being worn out by the constant pressure of these tumors on the pneumogastric nerve. Other members of the same family have fallen victims to the same disease.

*Calcareous Tumor.*

Dr. Enos showed a specimen of *calcareous tumor*, removed from the right eye-brow, and which had existed for seventeen or eighteen years, but which had not increased in size for the last ten. The tumor was quite moveable.

---

### REGULAR MEETING, JUNE, 1863.

*Pathological Specimens, by* Dr. Speir.

A woman, 67 years of age, fell on the side-walk, and was trampled upon by a team of horses. She died two days after the accident.

The post mortem revealed *emphysema of left side and neck*, *fracture of the scapula*, and of the *second, fourth and sixth ribs.* There was also a *double femoral hernia*, *fracture of the orbital plate of the frontal bone*, extending into the *petrous portion of the temporal bone*, and through the *sphenoid.* There was also *ecchymosis of the eyes*, an *atheromatous condition* of the *aorta*, and an *aneurism* at the *bifurcation* of the *iliac artery.*

### *Specimen of Pericarditis.*

The subject of this specimen was a man, 35 years of age, of intemperate habits. He had been on a spree for a week, and was attacked with pleuritis, when he entered the Brooklyn City Hospital. He died twenty-four hours afterwards.

On post mortem found eight ounces of serum in the pericardial sac, and coagulable lymph on its sides. The *liver* was fatty and atrophied, weighing only one and one-half pounds.

### *Specimen of Diffuse Popliteal Aneurism.*

The subject of this specimen was a colored man, who entered the Brooklyn City Hospital June 1st, 1863. On the 12th of January preceding, he had the left femoral artery tied for popliteal aneurism, the wound healing up by first intention. He then had an attack of articular rheumatism in the knee-joint, with considerable swelling. It was punctured with an exploring needle, and synovial fluid, with serum, flowed out. About the 1st of March, as he expressed himself, he felt something give way in the calf of his leg, as if a vein had burst; the blood gurgled up towards the knee, and looked like a mouse crawling under a sheet. The knee then swelled and became very painful. In the course of a few days it was opened by Dr. Raphael, and there was a discharge of grumous blood, resembling *meconium.* The articulation of the tibia and femur could be felt through the opening. This, it seems, has filled up, and been opened two or three times since, and when he entered the Brooklyn City Hospital it was discharging a bloody serum, intermingled with clots. The back of the leg felt boggy and undermined; the pulse was fair, appetite craving, mucous membrane pale. There was lateral motion at the knee-joint, and some crepitus. He complained of great tenderness along the back of the calf of the leg, and along the *tendo-achillis.*

On the 6th of June there was a free discharge of blood and serum, and on the next day some discharge of pus. Upon consultation by Drs. Kissam, Enos, Hutchison and Cochrane, *amputation* was decided upon, but the patient declined. Another opening was soon after made by the matter at the outside and back of the calf of the leg, which discharged a great deal of pus and blood. A bandage was then applied to the leg, from the toes upwards, which had to be occasionally removed, in consequence of its pressing too much on the *spine of the tibia.*

On the 11th of June, when the bandage was removed, there was a profuse arterial hemorrhage, not, however, *per saltem.* This was controlled by the persulphate of iron and bandaging.

On the 12th of June Dr.. Kissam amputated through the middle of the thigh, with very little loss of blood.

In examining the amputated limb, an aneurismal sac, about the size of a horse chestnut, was found ruptured in the popliteal space. This communicated with a large cavity, extending up and down among the tissues, and communicating with the cavity of the joint. The articular ends of the bones were partially deprived of cartilage. The femoral artery of the amputated limb was found atheromatous.

Dr. Bell remarked that in the case of the woman, the head was injured by the foot of the horse. She was perfectly conscious, and related everything that happened to her, stating that she had not been run over. There was no hemorrhage from the ears.

Dr. Enos thought that although the fracture of the head was so extensive, the displacement was so slight as not to produce any laceration of the *dura mater*, and hence the reason of no hemorrhage from the ear. In the case of pericarditis, he remarked that he had never seen so large a fibrinous deposit about the heart, and thought it very strange that life could have been prolonged, until such an extensive deposit was formed. He was also present at the amputation of the leg referred to, and did not notice any retraction of the vessels, nor did the skin seem to have any elasticity. The arteries had to be drawn out by the forceps, and there was very little loss of blood.

Dr. Wm. Gilfillan thought it a very remarkable case. He understood, from the history of the case, that the ligature was applied in January, and the aneurism did not appear until the following June. In this instance, therefore, the ligature must have, evidently, failed in curing the aneurism, as it became so diffused. He thought it a very singular circumstance, that after *ligation* the aneurism should have become *diffuse.* The circulation could not have been at any time cut off from the aneurism.

Dr. Enos stated that he had understood that the Doctor who operated at the time, did not attempt to obliterate the artery, but merely to cut off the circulation from the aneurism. He was present at the consultation, and it was very plain that there had been an erosion of the cartilage of the joint, as *crepitus* was very distinct. He could feel the pulsation of the artery, but could not say whether it was the *femoral* or the *profunda*, enlarged.

*Spiculæ of Bone from Wound.*

Dr. Enos presented several *spiculæ* of bone, removed from the tibia of a patient, the result of a gun-shot wound, received on the 6th of May, 1863. The ball entered below the head of the tibia, coming out on the other side, splitting and fracturing the bone. From time to time the pieces of bone were removed, and in one of these was found the nutrient artery. The patient, after a great deal of suffering from inflammation and suppuration, gradually recovered, and is now doing pretty well. The limb is shortened. The open and ulcerated surface was very painful, and morphine was introduced after the discharge of pus had stopped. Its application seemed to allay the pain, and produce active granulations.

*Fracture of the Skull with Depression.*

Dr. Wm. Gilfillan gave the history of a case of *fracture of the skull* with *depression*, and removal of the fragments. A private in the 1st Connecticut Artillery, was wounded in the head by the kick of a horse, on the 27th of June, 1862. No careful examination, it seems, had been made of the wound for some weeks. Water dressing was applied, and pus was freely secreted. He was discharged at Baltimore in September, and was admitted into the Long Island College Hospital on the 26th of the same month. On examination found a wound at the occipital protuberance, and on introducing the probe, the bone was felt denuded and rough, over a space of two or more inches. The probe, at one point, passed between the fragments, and touched the dura mater. The pus had the peculiar odor of dead bone, which, however, could not be moved by the probe. As separation must have taken place, after such a lapse of time from the receipt of the injury, it was decided to cut down and remove the dead pieces of bone. This was done on the 28th of September, and two pieces of bone were removed, exposing the dura mater for fully three inches by two. Considerable discharge of pus took place for two days afterwards. Two weeks subsequent to the operation, an abscess formed below the occipital protuberance, and was opened, discharging a large amount of fetid pus. He was discharged on the 20th of October.

In this case there were no symptoms of compression at any time, although the bone was depressed under the edge of the remaining sound piece of occipital bone. Nor was there any disturbance in the functions of the cerebellum. The soft parts healed readily over the cavity. But what was most singular was, that the inner table was completely gone, although the dura mater was fully exposed. He would ask, *what became of the inner table?* Was it dissolved in the pus, which was discharging freely for three months?

Dr. Bell remarked that the inner table must have been absorbed, or else lost in suppuration. In one piece *disintegration* seems to have gone on rapidly, as it is very rough, and pierced by a number of holes.

Dr. Enos thought that it could not have been lost in suppuration, as the inner table is very hard. We find bones, bathed in pus for months, remain intact, and do not become eroded. He thought, therefore, that it must be owing to absorption. It is a fact, that bone will sometimes break down, as the microscope will sometimes show this to be the case, in examining pus.

---

### QUARTERLY MEETING, JULY, 1863.

*Apothecaries and their relation to the Medical Profession.*

At a meeting of the Society, held July 14, 1863, the attention of the Society was called to the censurable course pursued by some of the druggists of this city. It was stated by several of the members that these gentlemen were in the habit of commenting upon prescriptions to the disparagement of physicians, substituting other articles for those prescribed, and not unfrequently renewing the prescriptions without the knowledge or consent of the attending physician, and that they were in the habit of prescribing for patients. For these, among other reasons, a committee was appointed, who made the following report, which was adopted:

*Whereas*, It is eminently desirable that the art of prescribing and dispensing medicines should conform, as far as possible, to scientific accuracy; therefore,

*Resolved*, That the Medical Society of the County of Kings recognizes the fact that physicians should be scrupulously careful in writing their prescriptions distinctly, and that they should use, as far as practicable, officinal names only.

*Resolved*, That it is the duty of dispensing apothecaries to put up prescriptions distinctly as directed, or to reject them, excepting, however, when there is cause to suspect a mistake; in which case, it is manifestly the duty of the apothecary to assure himself of the intention of the prescriber, before dispensing the prescription.

*Resolved*, That the practice which some apothecaries indulge in of treating cases of disease constitutes quackery in its worst form, because of the false confidence which their semi-professional character inspires in the minds of the people.

*Resolved*, That recommending nostrums, prescribing, criticizing prescriptions, or otherwise indulging in conversation tending to impair confidence in the author of a prescription, substituting other articles than those directed by physicians, keeping incompetent clerks, dispensing medicines of bad quality, repeating prescriptions against the expressed wish of the prescriber, and habitual carelessness, are all disreputable practices; and it shall be the duty of the members of this Society, who may hereafter become cognizant of such conduct, to report the same to the Society for the benefit of his fellows.

*Resolved*, That these resolutions and preamble be approved by the President and Secretary in behalf of the Society, and published; and that a copy of the same be presented to every apothecary in the county, if practicable.

---

### QUARTERLY MEETING, OCTOBER, 1863.

### *Specimen of Entozoa.*

Dr. Speir presented an interesting specimen of entozoa, taken from the right side of the heart of a dog. In the evening the animal was in perfect health, but on the following morning was found dead. On opening the heart these *worms* were found (dead) in the right ventricle.

In reply to a question the Doctor stated that he had never found these animals in the human heart, but had discovered them in the kidneys. In this ventricle two were found, besides a clot of blood. The question here recurred, how did they get into the ventricle?

Dr. Enos looked upon it as a very interesting subject, and well worthy of being thoroughly sifted.

*Pyemia.*

Dr. Speir also presented a specimen of pyemia. The subject was a man who recently died in the Brooklyn City Hospital. He had been operated upon a short time previously for a compound fracture of the leg. Gangrene set in, in the stump, and the man died. On post mortem, found the blood vessels, especially the superficial veins, distended with air. The blood was also *oily*, which seemed under the microscope, to be principally *margarine.* The liver was waxy, but the other organs of the body appeared to be healthy. Air was also found in the cephalic vein.

---

### REGULAR MEETING, OCTOBER, 1863.

*Hypertrophy of the Heart.*

Dr. Ball presented a pathological specimen of hypertrophy of the heart. The subject, a man 35 years of age, had had, when quite young, several attacks of rheumatism. In examining the heart, found a dilatation, and disease of the valves. The valves of the pulmonary artery seemed to be normal, but in the others were found large deposits of calcareous matter. The weight of the heart was twenty-five ounces avoirdupois. Another remarkable characteristic of this case was an undescended and undeveloped testicle, the other being perfectly normal.

*Cystic Tumor of the Breast.*

Dr. Ball also presented another specimen, which he deemed very important in point of diagnosis, viz: a diseased mammary gland.

On the 12th October, 1863, was called to see an unmarried woman, aged 40 years, suffering from a tumor in the left breast. It had the *feel* of scirrhus, although the history of the case would not induce that belief. It was rapidly growing larger, however, and produced a great deal of uneasiness and trouble. Told her that it was not of a malignant character, and advised its removal. She consented, and it was removed on the 16th instant. On examination it proved to be a cystic tumor, with two cysts, one of which would contain an ounce. The fluid was serous, and of a brownish color. He thought it a very interesting case in regard to forming a diagnosis between it and scirrhus.

In reply to a question, Dr. Ball stated, that in the case first mentioned, the man had suffered a great deal from cardiac trouble, and when last seen he was suffering from great dyspnœa, produced by hydro-thorax and hydrops-pericardii. In regard to the undeveloped testicle he would remark, that the man was the father of three or four children.

Dr. Enos stated that another point of interest in the case was an inguinal hernia, on the same side with the undeveloped testicle. In regard to the diseased mammary gland, he would state, that the two cysts occupied the posterior part of the breast, between the cellular tissue and the adjacent parts. They were not lined by epithelium. The fluid was serum, and contained more or less oil globules; but these had, undoubtedly, floated out from the surrounding structure. Whether the disease had originated in the lactiferous tubes or not, he could not say; but he was inclined to think that it did not, judging from the position of the tumor. It was not a compound cyst, nor of a cancerous nature.

Dr. Jones desired to know, what advantage could accrue to the patient in removing the breast, and also whether benign tumors of the mammary gland, of the character represented, would lead, in the end, to malignant trouble.

Dr. Ball replied, that when he first saw the tumor it was large, and seemed to be increasing very rapidly, producing great inconvenience to the patient. To obviate this was one great advantage in its removal. He doubted whether the disease would ever degenerate into scirrhus.

Dr. Ford had often seen tumors of the breast assume a considerable size, and give a great deal of trouble; but the difficulty soon subsided, and the tumor entirely disappeared. He thought, therefore, that it would have been more judicious to have waited, and practiced conservative surgery.

Dr. Jones had had two patients with tumors of the breast, which at times, increased rapidly, and as often decreased, causing no detriment to their health. In one of these cases he thought of removing the breast, but did not feel justified in doing so. His own opinion in regard to Dr. Ball's case was, that the operation might have been deferred.

Dr. Gardiner called the attention of the Society to the use of the compressed sponge in tumors of this character, a subject which was discussed, some time ago, at one of our monthly meetings. He had had a case of enlarged cervical glands, which he had treated successfully with compressed sponge. In the treatment of benign tumors of the mammary gland, Dr. Batchelder used this means with great efficacy.

Dr. Ball thought it proper to state, that he never advised operation in these cases, when the tumor was stationary; but in the present instance it was developing so rapidly that he thought its removal justifiable.

Dr. Speir presented several pathological specimens from post mortems recently held at the Brooklyn City Hospital.

*Fracture of the Liver.*

This specimen was from a boy who had been run over by a swill cart, and who died about an hour afterward. The lesions in this case were a fracture of the liver through its longitudinal fissure, and a partial fracture of the kidneys. The abdomen was nearly filled with blood, and the heart contracted, but no clots were found in its cavities. There were tuberculous deposits in the lungs, and the thymus gland was undergoing cystic degeneration.

*Tuberculosis.*

Another specimen was taken from a man, 40 years of age, who was admitted about two months before death, in the hospital, for chronic diarrhœa. He complained exceedingly of shooting pains across the bowels. He failed rapidly and died on the 17th instant.

The lungs contained deposits of arrested tubercles; the liver was softened and tuberculous, containing an abscess, which communicated with the gall-bladder. The kidneys were fatty, and in the left one was found a small renal calculus. The rectum was ulcerated, and contained a tuberculous deposit. The diaphragm was adherent to the liver in several places. The *matter* from the abscess was examined under the microscope, and found to contain tubercles. The cause of death was tuberculosis of the liver.

In reply to a question, Dr. Speir stated, that the boy had all the external marks of injury about the region of the liver. About three pints of blood were found in the cavity of the abdomen.

Some discussion here ensued in reference to abscess of the liver, Dr. Enos remarking, that while in Paris he saw a case of the kind, where the *matter* was discharging through the hypochondriac region, the attending surgeon having a long tube inserted, to facilitate its discharge.

Dr. Jones had treated one of these cases, and supposed it, at the time, to be an acute inflammation of that organ. When the patient was able to go about, he went to New London, where he was again taken sick. He (Dr. Jones) was subsequently informed that the person had had an abscess of the liver, which discharged freely, and he recovered.

---

### REGULAR MEETING, NOVEMBER, 1863.

### *Therapeutics.*

Dr. C. L. Mitchell called attention to his recent experience in the effects of certain remedies.

1.—Quinine, which for the last year he had found remarkably beneficial in allaying bronchial irritation, especially in children. He has used it in some cases of almost incessant cough, and had found it successful after the failure of all the more usual anodyne expectorant remedies. In the bronchial irritation of pneumonia he had found it equally effectual and reliable. He cited the case of a child, aged 11, with excessive bronchial irritation, causing almost incessant cough, following measles. Skin dry, pulse 120, anorexia and extreme feebleness. A grain of quinine every two hours, was followed by immediately beneficial effects, and a speedy recovery.

A case of hay asthma was last year much benefited, and this year wholly overcome, by the use of quinine. He also alluded to the good effects of quinine in infantile convulsions, previously reported; and to its almost specific effects in neuralgia, especially in cases of miasmatic origin. He had recently had a most inveterate case, that had resisted other remedies, to whom he prescribed three grains every two or three hours, subsequently five grains, and finally ten every hour, which effectually subdued the disease, without any very unpleasant effects. There was a little tinnitus aurium, and decided sedative effects—lessened frequency of the pulse—and quiet sleep, of which the patient was in great need.

Dr. McClellan's experiences in the use of quinine for the last year or two confirmed that of Dr. Mitchell. He frequently prescribed it in bronchial irritation following measles or other causes, and considered it a remedy of great value in such cases. He had recently used it in two cases of children, with persistent barking cough, with perfect relief. In these cases he prescribed two grains four times a day.

Dr. Reese, *per contra*, cited the case of a lady who had been subject to neuralgia for twenty years, generally in the head and face, but recently of the sciatic nerve, to whom, in accordance with the counsel of Dr. Mitchell, he had given ten grains of quinine every two hours, until seventy grains had been taken, without benefit. On inquiry he found that this patient had been benefited in previous attacks by visiting Islip, L. I., was therefore interested to find out the peculiarities of the place—whether it was in reality "going to the pines."

Dr. Mitchell was gratified at the mention of this case by Dr. Reese. It served to show the necessity of constant research and observation in the effects of remedies, no less than exceptional cases to our best experience. Dr. Mitchell also mentioned other remedies, but their consideration was deferred to another meeting.

### *Phlegmasia Alba.*

Dr. McClellan reported an unusual case of *phlegmasia alba*, recurring in the fourth week of a fatal case of typhoid fever. It began in the posterior part of the left thigh, extending to the leg and foot, and finally the whole extremity, but not following the course of the veins. The patient was improving under the local application of camphorated oil and cotton, and the internal use of iron and quinine.

Dr. Mitchell had seen somewhat similar sequelæ of fever in males, but none recently. He had also seen apparently, very perfect phlegmasia alba in persons predisposed to erysipelas. He cited the case of a lady, involving both lower extremities, finally extending to the lower part of the abdomen; resulting in deep cellulitis, abscess and death. Toward the last, however, the disease assumed a more erysipelatous character. The patient was for a time benefited by the use of quinine and iron.

### *Granular Disease of the Kidney.*

Dr. Bell reported a case of *Bright's Disease*, relieved by the use of *bitartrate of potash.*

Catharine Nevins, widow, aged 33, was admitted to the Brooklyn City Hospital, August 19th. She had been sick for several months, and for the last three weeks under the care of a physician. She was, at the time of her admission, suffering with severe ptyalism, had general anasarca and ascites. Complained of general lassitude, headache, pain in right hypochondrium and epigastrium, and frequent nausea. Had been purged, but for last two or three days constipated. She was ordered half an ounce of compound jalap powder, which operated well. On examination of the urine next day, she having passed twenty-seven ounces, it was found to be, s. g. 1009, to contain about 20 per cent. of albumen, a large quantity of granule cells, fatty tube casts, and triple phosphates.

She was directed to take one drachm of bitartrate of potash three times daily. Within a week the quantity of urine passed increased to four quarts during the twenty-four hours, with alleviation of all the most urgent symptoms. The bitartrate of potash, serving the double purpose of being an efficient diuretic, and sufficiently active aperient. The quantity of albumen gradually lessened until there was barely a trace—September 16th—and anasarca and acites totally disappeared. At the last examination of the urine, microscopically, October 3d, no tube casts or granules were discoverable.

The bitartrate of potash was discontinued September 16th, and she was put upon pyrophosphate of iron with tincture of quassia as a tonic, up to the time of her discharge, relieved, October 5th. Dr. Bell has seen the patient once since, two weeks ago, and there is as yet no re-appearance of the disease.

The suggestion for the use of bitartrate of potash in *Bright's Disease*, Dr. Bell accredited to Bennett.

Dr. Mitchell said he used the bitartrate in cases of dropsy after scarlet fever, but in Bright's Disease he had generally relied upon corrosive sublimate.

Dr. McClellan usually associated local depletion over the region of the kidneys with the bitartrate.

Dr. Enos inquired if the members of the Society had found corrosive sublimate to produce salivation in such cases; and stated that he recalled a case in which he administered it to a patient who had been sick four or five years, suffering from swelling of the limbs and abdomen, accompanied with albuminuria. Drs. Parker and Detmold saw the patient and pronounced it incurable. He (Dr. E.) gave corrosive sublimate in one-eighth grain doses, which produced salivation. The patient recovered notwithstanding.

Dr. Jones remarked in regard to the use of mercurials in Bright's Disease, that some time ago he had a case under his care, but the patient not recovering rapidly enough to satisfy the friends, after two weeks' attendance he was discharged and another physician called in. The patient was then put upon mercurials and soon after died. He discontinued the use of mercurials in such cases.

Dr. Enos stated that the case mentioned by him happened years ago, when he first began to practice medicine. In this instance he had given the mercurial, and he heard afterwards that the patient had recovered. Six or eight years subsequently the disease returned. His own belief in this respect was, that neither mercurials or anything else would cure a well developed Bright's Disease.

### *Catarrh-Inhalation.*

Dr. Landon reported two cases of chronic catarrh successfully treated by the inhalation of tinc. Bensoin comp. In one of these cases various remedies had been used without benefit for several months. Tinc. Bensoine comp. was then used by inhalation by means of an inhaler fitted to the nostrils, the patient was relieved at once, and in a short time, well. The second case was of six years' standing, accompanied by a very offensive discharge from the nose. One drachm of the Tr. Benzoin Comp. diluted with from ℥i to ℥iss of hot water was inhaled, ten or fifteen minutes at a time, with the same result as in the first case.

Dr. Mitchell stated that he had met with good success from the injection of cold water, and also of sage tea in such cases.

Dr. Ford had good results from nitrate of silver, grs. 15 to the ʒi.

Dr. Bell used an anodyne usually of compound solution of opium at night; and Dr. Clark, quinia.

*Turpentine in Chorea.*

Dr. Crane asked the experience of the members in regard to the use of *turpentine in the treatment of chorea.*

Dr. McClellan related two cases which he had treated with turpentine since hearing it spoken of by Dr. Crane. The first case was of two years' duration, in a child six years of age; turpentine was at first given in cathartic doses with marked benefit, and afterwards fifteen drops three times a day for a fortnight, when the chorea ceased. The second case came under his observation one month ago—was treated with small doses of turpentine with same result.

Dr. Crane reported a case of strongly marked chorea, a girl, aged 10; this was an acute case. The patient was first treated with mercurial cathartics and carb. ferri. She grew worse and worse, becoming idiotic, with loss of the power of locomotion and articulation, and the irregular motions persisted during sleep. He then gave two teaspoonfuls of turpentine on account of the mother's suspicion of worms. No worms were passed, but the patient's symptoms were relieved, and three days after the dose was repeated, with the same results. This was continued twice a week with marked benefit, for a month or six weeks, when the child was completely relieved, and there has been no recurrence of the disease.

Dr. Landon stated that he had treated a case of obscure brain disease, accompanied by convulsions and insensibility, with small doses of turpentine, with a very favorable result, and it served to illustrate in his mind the utility of the remedy in chorea, though he had never before thought of it in this relation.

*Removal of foreign bodies from the Nostrils.*

Dr. Enos exhibited a bean which was removed from the nose of a child about five years of age. He did so for the purpose of speaking of a simple mode of removing foreign bodies from the nostrils—a mode which, though not mentioned in the books, he has found very easy and efficacious. He would call it the blowing process. The manner is this: the child being in the upright position, place your mouth upon the *open* mouth of the child, and while with one finger you strongly compress the unobstructed nostril, blow somewhat forcibly and repeatedly till the foreign body is forced out. The rationale of course is simple. When the air passages of the child are filled the column of air mounts over the velum into the nares, and impinges upon the posterior surface of the obstructing body, and forces it forward. Sometimes it will be forcibly ejected several yards.

*Vesico-Renal Calculus.*

Dr. Speir presented a specimen of vesico-renal calculus, with the following history of the case, written by Dr. Mitchell, the attending physician:

"First saw the patient, about six years ago, in consultation with the late Dr. Brooks. She had been suffering from, what she called, strangury, since 1848, the pain of which had become so aggravated, that in 1853, when Dr. Brooks was first called in, she had been confined to her bed for four months. Since that time, with the exception of a short time in 1854, she has been unable to be in the upright or sitting position without extreme suffering. Previous to 1853, she had been attended by Dr. Clinton of New York, assisted by one of the most eminent surgeons of the city, Dr. Hoffman. The symptoms have been nearly the same since the beginning of the attack, viz: a constant and painful desire to make water, attended with violent straining and bearing down pain, resulting in the discharge of a few drops, or perhaps a single drop of urine, at a time. The distress was increased, at times, in spasms, during which the pain was insupportable. Minute calculi were occasionally passed. No relief was obtained, except by the use of opium. In 1859 she came under the care of Dr. Simons of New York, having been removed for that purpose to the Woman's Hospital. Some relief was afforded by making an opening through the vesico-vaginal septum, but the relief was of short duration, owing to the immediate closure of the orifice. A repetition of the operation was followed by similar relief, and an equally rapid healing of the incision. The opening closed up, even when produced by laceration. Dr. Chapman visited her with me in November, 1862, at which time the same state of things were continuing, the operation on the vesico-vaginal septum having been relinquished after the first three attempts, three years previously. The patient was put under the influence of ether, and the catheter introduced, showing that there was no permanent stricture of the urethra, nor any stone in the bladder. The same operation was subsequently repeated, and with a like result. The suspicion of vesical calculus was entertained by every physican, I believe, who saw the patient, but none was discovered by Drs. Hoffman, Simons, Emmet, Chapman, or myself, although each had examined the bladder, with a view to diagnosis. There had been little or nothing to direct attention to the kidneys. The case was supposed to be one of inflammation of the bladder, with irritable urethra. During the last year there has been, at times, pain in the region of the kidneys, but not to a degree to attract much of the patient's attention, as I am informed by the daughter, who nursed her. Since November, 1862, there has been no physician in attendance."

At the post mortem Dr. Speir found the following lesions: The liver was large and fatty, firm, and of a yellowish color. The kidneys were fatty, and the capsules firmly adherent. In the pelvis of the kidneys were found several renal calculi. In the bladder was also found a calculus, two inches long, and one inch wide; it was free, and composed of the triple phosphates. The walls of the bladder were indurated, and the mucous membrane ecchymosed and inflamed.

Dr. Jones thought it would be very interesting to know how long it took the triple phosphates to form.

In reply to different inquiries, Dr. Hallet stated, that it was the opinion of the attending surgeon, that no calculus was present six years ago. About two years since, however, minute calculi were found in the urine; but these particles, when first passed, did not look like calculi, and it was only after exposure to the air that they became quite hard.

Dr. Hutchison remarked that if any calculus was present in the bladder, at the time of the operation, Dr. Simons would, undoubtedly, have detected it. It was a very interesting case in every point, and at the same time so important that he would advise a reporter to be appointed to hunt up all the facts possible in regard to it. A resolution to that effect having been subsequently offered, it was adopted unanimously.

*Inflammation at the base of the Brain—without Delirium or Convulsion—Death.*

Dr. Enos reported the following case:

Mrs. P——, aged 37, mother of five children, the youngest five months, at the breast, was taken with a chill on Monday, December 1st. The rigor was *very severe*, followed by intense headache *all over the head.* This was much aggravated by motion. Mind clear, light a little painful. She had not slept for several nights on account of the illness of one of her children. A teaspoonful of laudanum injected into the rectum, and a warm pediluvium abated the pain for a time, but it returned again the next day, and was so severe that fearing inflammation, leeches were applied to the temple and behind the ear. This relieved the pain. Bowels were moved by a saline cathartic; pulse not much accelerated; mind clear. As the pain *seemed* to be neuralgic, pills of hyos. and camph. were given when the pain returned, but without relief. Laudanum injection again allayed it. Thursday she had most intense pain in her back and limbs, which continued till the laudanum injection was again given. Friday she appeared better, ate a little, slept some. Saturday, at 4 A. M. had *acute pain in her head and back.* The injection again eased her till 7 o'clock. At this time she was powerless, could not move hand or foot, but spoke distinctly, no great pain, respiration 20, loud tracheal rales, pulse full, strong, 120; *pupils contracted;* distended bladder, relieved by catheter; speech became more and more difficult, not unlike the lisp of intoxication; mucus foamed out of the mouth; *mind continued clear;* inability to swallow came on, and she died easily at 2 P. M. Death commencing at the lungs.

*Post Mortem.*—Cerebral vessels somewhat congested; pus between the arachnoid and pia mater, over the medulla oblongata, cerebellum, pons varollii, and a little upon the summit of the right cerebrum; choroid plexus dropsical, but little fluid in the ventricles. The state of the mind led us to locate the difficulty at the base of the brain. Hence the severe pain in the back and limbs and final paralysis; the interference with the function of the pneumogastric nerve, and death beginning at the lungs.

---

### REGULAR MEETING, DECEMBER, 1863.

### *Uremia.*

Dr. Minor exhibited a *stone,* taken from the bladder of a man who had been suffering, for a long time, from the usual symptoms, accompanying this complaint. On post mortem found a great amount of disease, the *bladder, ureters,* and *kidneys* being alike involved. In opening the kidneys a quantity of sero-purulent matter flowed out; the uriniferous cones were mostly absorbed, and but a small part of the secreting portion was available for service. The left kidney was mostly affected. The ureters were extensively dilated throughout their length, the mucus membrane being smooth and glassy, and duplicated about two-thirds of the way down. The mucous membrane of the bladder was thickened, and undergoing inflammation. During life-time a large *sound* was introduced without difficulty, thus demonstrating the fact of the non-existence of a *stricture.* The question here recurred, where did this disease begin? In his own opinion it commenced in the bladder, the stone producing a chronic inflammation of that organ, which passed up the ureters, and finally into the kidneys. The stone obstructing the orifice, prevented the flow of water into the bladder, and the ureters became dilated. It is impossible to understand how this man could live as long as he did with such an amount of disease. The man died of uremia. The stone was composed of the oxalate of lime and the triple phosphates.

Dr. Jones desired to know, whether the same thing would occur in this, as in some other organs of the body; that if one kidney is diseased, the other will increase in size, and do double duty.

Dr. Minor was aware that the lung, in certain cases, would perform an extra amount of duty, but it was difficult to determine whether the kidney would do the same thing. In this instance both kidneys were involved in the disease.

Dr. Squibb stated that there was evidently, in this case, two diatheses, as the composition of the stone abundantly proved. The disease was, undoubtedly, owing to a mechanical obstruction.

In reply to a question, Dr. Minor stated, that there might have been a *temporary* stricture, but not a *mechanical* one, as the bladder did not become largely distended. He thought that a man, with such a stone in his bladder, might be compared to a Davidson's syringe. The stone first blocked up the orifice, and as soon as dilatation occurred, the stone rolled back, and thus allowed the urine to pass out. He knew of a similar case to the one reported by Dr. Minor, where the bladder became sacculated.

Dr. Minor stated that Dr. Speir had examined the kidneys, and could find but little of the excreting portion left.

Dr. Hutchison remarked that he made a post mortem some years ago, where he found the *ureters* and *kidneys* very much distended. He presented the specimen to the Pathological Society of New York, and Dr. Alonzo Clark expressed the opinion that the cortical portion was not destroyed, although appearances indicated it.

### *Pyæmia.*

Dr. Hutchison presented several specimens of diseased organs, taken from a subject 60 years of age. About nine months ago he was called to see this patient, and found him suffering from retention of urine. Introduced a No. 9 catheter, with very little difficulty, but detected no organic stricture, or the presence of a stone. On inquiry found that for the last four years he had noticed an irregularity in the stream of water; it was sometimes twisted, at other times forked. He had had also, some difficulty, at times, in getting his urine started; it would then stop, and start again. The Doctor did not see him again until about ten days before his death, when he stated that he had recovered somewhat; although he had to strain a great deal to urinate. At this visit detected a hernia on the right side. On the 28th November passed a No. 10 sound, but could detect no stone. On the 29th he passed his urine freely, but on the 30th he again complained a great deal, the stream of water being very small. This continued up to the time of his death, December 8th. On examining the urine two weeks ago, found it to contain pus. During the last three or four days of his life he complained of severe *rigors*, followed by sweats and unconsciousness. The treatment consisted, mainly, of stimulants and soothing remedies.

On post mortem, found the *lungs* tuberculous; both lower lobes were greatly congested, but showed no evidence of inflammatory action. He had had no cough. The heart and appendages were healthy, with the exception of an atheromatous patch in the ascending portion of the aorta. The liver was also healthy. In the kidneys were found patches of tubercles, scattered throughout its substance; the pelvis was distended with purulent fluid. The ureters were enlarged, and had a valvular arrangement along their course. The left kidney was in a pretty good condition. In the bladder were found several abscesses, some of which extended entirely through the peritoneal coat. These abscesses seemed to be in the muscular tissue of the organ. The mucous membrane was thickened, although at the neck of the bladder it was normal. The third lobe of the prostate gland was enlarged, and what was peculiar in this case was, that there were symptoms of *stricture*, when in reality, no obstruction existed. This, however, may have been owing to the condition of the gland referred to. The specimens were examined, and the tubercles found, as represented. The pulse was 84. The cause of death may have been pyæmia, or formation of abscess in the bladder; but in such cases the pulse is usually very frequent.

Dr. Minor felt very much interested in this case, and thought that the difficulty in micturition was owing to some spasmodic action. In his opinion the symptoms seemed to indicate *pyæmia*.

Dr. Enos thought that no other explanation could be given for the difficulty in micturition, than that advanced by Dr. Hutchison, viz: enlarged prostrate gland.

